# Recombinant Adenoviruses for Delivery of Therapeutics Following Spinal Cord Injury

**DOI:** 10.3389/fphar.2021.777628

**Published:** 2022-01-10

**Authors:** Anastasiia O. Sosnovtseva, Olga V. Stepanova, Aleksei A. Stepanenko, Anastasia D. Voronova, Andrey V. Chadin, Marat P. Valikhov, Vladimir P. Chekhonin

**Affiliations:** ^1^ Department of Fundamental and Applied Neurobiology, V.P. Serbsky National Medical Research Center of Psychiatry and Narcology, The Ministry of Health of the Russian Federation, Moscow, Russia; ^2^ Department of Neurohumoral and Immunological Research, National Medical Research Center of Cardiology, The Ministry of Health of the Russian Federation, Moscow, Russia; ^3^ Department of Medical Nanobiotechnology, Institute of Translational Medicine, N.I. Pirogov Russian National Research Medical University, The Ministry of Health of the Russian Federation, Moscow, Russia

**Keywords:** recombinant adenovirus, spinal cord injury (SCI), neurotrophin 3 (NT3), brain-derived neurotrophic factor (BDNF), glial cell derived neurotrophic factor (GDNF)

## Abstract

The regeneration of nerve tissue after spinal cord injury is a complex and poorly understood process. Medication and surgery are not very effective treatments for patients with spinal cord injuries. Gene therapy is a popular approach for the treatment of such patients. The delivery of therapeutic genes is carried out in a variety of ways, such as direct injection of therapeutic vectors at the site of injury, retrograde delivery of vectors, and *ex vivo* therapy using various cells. Recombinant adenoviruses are often used as vectors for gene transfer. This review discusses the advantages, limitations and prospects of adenovectors in spinal cord injury therapy.

## Introduction

Spinal cord injury (SCI) is serious medical condition, often leading to disability and a significant decrease in patients’ quality of life. Unfortunately, existing therapies for SCI are ineffective and do not lead to complete or significant functional recovery (Tator, 2006; Bydon et al., 2014; Tsintou et al., 2015). Numerous ascending and descending nerve pathways are interrupted after SCI, causing a partial or complete impairment of sensory and motor functions below the injured spinal cord (Lin et al., 2016). The recovery of large regions of damaged tissue in the spinal cord or brain requires the growth of axons either by compensatory growth of preserved fibers or by the regeneration of damaged axons (Maier and Schwab, 2006). The inability of the central nervous system to regenerate is explained by the presence of inhibitory molecules, the lack of supporting molecules, the presence of cell barriers, and the inability of adult neurons to support axonal growth over long distances (Silver and Miller, 2004). SCI therapy aims to prevent neuronal death and stimulate axonal regeneration (Blits et al., 2000).

The use of neurotrophic factors is an actively developing direction for the treatment of SCI, since they are able to suppress neuronal apoptosis and support atrophic, hypofunctional neurons. These factors quickly degrade after direct administration. Gene transfer that permits the stable production of neurotrophic factors is being actively studied (Betz et al., 2016). Recombinant adenoviruses (rAdVs), adeno-associated viruses (AAVs) and lentiviruses are the main vectors being studied for transgene delivery; each one has its own advantages and disadvantages. The use of AAVs and rADVs is safer due to episomal transgene expression (Lee et al., 2017). Furthermore, an important difference between them is that AAVs and lentiviruses cause constant expression of a transgene, while rAdVs provide only temporary expression (Tosolini and Morris, 2016). Neurotrophic factors can alter the dendritic architecture, synaptic density and plasticity (Horch, 2004; Kuipers and Bramham, 2006; Parrish et al., 2007; Rodger et al., 2012). There is scanty of data on how the long-term expression of secreted neurotrophic factors affects the structure and function of neurons. For instance, the transplantation of an autologous peripheral nerve graft transduced with AAV producing brain-derived neurotrophic factor (BDNF) or ciliary neurotrophic factor (CNTF) led to significant changes in dendritic architecture in both transduced and non-transduced populations of regenerating retinal ganglion cells after 5–8 months (Rodger et al., 2012). The long-term expression of BDNF was found to induce spasticity and hyperexcitability (Fouad et al., 2013), while its transient expression was sufficient to maintain the regenerated axons for long periods of time at the sites of SCI (Blesch and Tuszynski, 2007). This review discusses the preclinical data on the use of rAdVs as vectors to deliver therapeutic transgenes after SCI.

## Three Generations of Recombinant Adenoviral Vectors

Vectors based on adenovirus serotype 5 (AdV5) have found a broad range of applications in the field of gene therapy, including the transfer of various therapeutic transgenes for the treatment of SCI. rAdVs have several advantages over other viruses used for gene transfer. They are able to transduce almost any cell type, including postmitotic cells such as neurons while providing a high level of transgene production when strong promoters such as CMV or CAG are used. In addition, rAdV5 has a high packing capacity, which may reach up to 36 kb with the removal of viral genes, and highly purified adenoviral preparations can be obtained at a high titer (Liu et al., 1997; Danthinne and Imperiale, 2000; Alba et al., 2005; Blits and Bunge, 2006).

There are three generations of rAdVs. First-generation adenoviral vectors lack the E1 and often the E3 regions of the genome with a packaging capacity up to 8 kb. Removal of the E1 region leads to almost complete prevention of viral replication and cell lysis, which makes these vectors relatively safe for gene therapy (Hermens and Verhaagen, 1998; Danthinne and Imperiale, 2000; Blits and Bunge, 2006). However, first-generation rAdVs elicit a significant immune response *in vivo*, mainly due to leaky production of viral proteins in association with a relatively rapid decline in transgene expression (after approximately 2 weeks) (Hermens and Verhaagen, 1998; Danthinne and Imperiale, 2000; St George, 2003; Blits and Bunge, 2006; Crystal, 2014; Lee et al., 2017). Despite this, first-generation rAdVs remain an invaluable gene transfer tool. Their packing capacity is sufficient in most cases, and they are easy to design and produce in large quantities. Most researchers in the field of SCI therapy use first-generation rAdVs, and much of this review will be based on the data regarding these vectors.

Second-generation rAdVs have deletions in the E2 and E4 regions, along with deletions in the E1 and E3 regions of the genome. These modifications increase the packing capacity to 14 kb and reduce cytotoxicity. Deletion of the E2A region increases the expression time of transgenes up to several months by preventing the synthesis of late viral proteins (St George, 2003; Crystal, 2014). However, second-generation rAdVs also trigger immune responses that lead to a decrease in the number of transduced cells (Crystal, 2014).

Third-generation rAdVs, also known as “gutless” or “helper-dependent” adenoviruses, have most of the viral genes removed. They retain only the *cis*-elements necessary for the replication of viral DNA and the packaging of DNA into a capsid. The development of such vectors requires the presence of helper viruses in the cell, which ensure the presence of virus-specific proteins necessary for viral replication and the assembly of adenoviral vector particles. Theoretically, third-generation rAdVs can contain several transgenes with a total size of approximately 36 kb. The injection of third-generation rAdVs into the body does not cause a significant adaptive immune response (Alba et al., 2005) correlated with the long period of transgene expression (up to 2–3 years) after a single injection (Toietta et al., 2005; Brunetti-Pierri et al., 2009). However, the production of third-generation rAdVs is complex and requires optimization (St George, 2003; Crystal, 2014; Lee et al., 2017).

## Delivery Routes for Recombinant Adenoviruses

There are several ways of delivering rAdVs with therapeutic transgenes into the damaged spinal cord. For example, there are direct methods of delivery, such as injection of rAdVs into the site of damage to the spinal cord or nearby intracerebroventricular, intraparenchymal or intrathecal administration using a catheter, minipump or syringe. Transgene expression was detected in several segments rostral and caudal to the injection site (2–6 mm) in both white and gray matter upon direct injection of rAdV5 carrying different reporter transgenes into the spinal cord after injury (Hermens and Verhaagen, 1997; Liu et al., 1997; Huber et al., 2000; Tai et al., 2003; Wang J. M. et al., 2011; Povysheva et al., 2017). Although these methods result in transgene expression, they are quite invasive. There are risks of spreading traumatic damage and worsening the nervous damage, including necrosis and apoptosis (Hendriks et al., 2004; Nakajima et al., 2005; Tosolini and Morris, 2016). In addition, these methods do not promote targeted synaptogenesis between severed axons and motor neurons.

There are also indirect delivery methods. For example, targeted retrograde gene delivery by rAdVs through the peripheral nervous system or through intramuscular injection is used to reduce invasiveness in rodent models. The intramuscularly injected rAdVs are captured by the neuromuscular junction via the presynaptic axon and are retrogradely transported along peripheral nerves to the corresponding motor neurons of the spinal cord (Tosolini and Morris, 2016). Upon retrograde delivery of an rAdV expressing GFP, the transgene was distributed along the rostral spinal cord in motor neurons of the anterior horns and was found in interneurons within the gray matter near the central canal of the injured spinal cord (Zhou and Shine, 2003). The locations of the motor end plates for the major muscle groups of the forelimbs and hindlimbs have been mapped in rats and mice (Tosolini and Morris, 2012; Tosolini et al., 2013; Mohan et al., 2014, 2015). Thus, targeted delivery of genes through the peripheral nerves or target muscles may be less invasive than direct injection and has the advantage of allowing repeated administration (Romero et al., 2000; Nakajima et al., 2005).

*Ex vivo* gene therapy is another class of indirect gene transfer techniques. This gene therapy includes obtaining the cells from the host organism, genetically modifying these cells *in vitro*, determining the expression level of the transgene in the modified cells, and then transplanting the cells back to the host. Different types of cells, such as fibroblasts, Schwann cells, and various stem cells, may be used for gene therapy aimed at restoring the nervous system. The risk of immunological rejection is reduced through the use of autologous cells. Thus, this approach has the ability to provide localized delivery of therapeutic transgenes to the site of injury and long-term, high-level expression of those genes at the target site (Jones et al., 2001; Hendriks et al., 2004).

## The Transduction Efficiency of Recombinant Adenoviruses

A direct injection of rAdV into the spinal cord leads to the transduction of neurons (Hermens and Verhaagen, 1997; Liu et al., 1997, 2010; Huber et al., 2000; Miura et al., 2000; Koda et al., 2004); astrocytes (Liu et al., 1997; Huber et al., 2000; Miura et al., 2000; Tai et al., 2003), including reactive astrocytes (Huber et al., 2000; Chen et al., 2016); oligodendrocytes (Hermens and Verhaagen, 1997; Huber et al., 2000; Tai et al., 2003; Liu et al., 2010); macrophages; lymphocytes; and microglia (Liu et al., 1997; Abdellatif et al., 2006). At the same time, several studies have shown that the main pool of transduced cells is represented by astroglial cells (Hermens and Verhaagen, 1997, 1998; Tai et al., 2003; Abdellatif et al., 2006; Liu et al., 2010). Peak expression was observed after 7–10 days (Huber et al., 2000; Koda et al., 2004; Povysheva et al., 2017), while practically no stained cells remained at the injection site after 2 months. Expression of a reporter transgene at a distance from the injection site persisted in neurons for at least 2 months (Liu et al., 1997). In contrast, other works have shown that long-term transgene expression is maintained by glial cells (Hermens and Verhaagen, 1998; Tai et al., 2003).

The cells transduced in the healthy spinal cord by the retrograde administration of rAdV were observed mainly in the gray matter and included motor neurons and interneurons, with peak transgene expression occurring on the seventh day after administration; a decrease in transgene expression was observed after 2 weeks (Nakajima et al., 2005, 2007). In the injured spinal cord, cells transduced by an rAdV expressing β-galactosidase were found in both gray and white matter (Baumgartner and Shine, 1998; Nakajima et al., 2005, 2007). A high level of transgene expression was observed in neural cells for 3 weeks (Zhou and Shine, 2003). In twy/twy mice with spontaneous chronic mechanical compression, retrograde delivery of rAdV to the spinal cord led to transduction of both neurons and glial cells, including oligodendrocytes (Uchida et al., 2008, 2012). Data on the cellular tropism of adenoviral vectors on base Ad5 after direct and retrograde administration healthy animals or after SCI are presented in Table 1.

**TABLE 1:** Cellular tropism of adenoviral vectors on base Ad5 after direct and retrograde administration in spinal cord.

Delivery routes	Tropism	Model of SCI	Animals	Reference
Direct injection	Neurons	Non-injured	*Rat*	Liu et al. (1997), (2010); Hermens and Verhaagen, (1997)
		Contusion	*Rat*	Liu et al. (2010); Chen et al. (2016)
		Transection	*Rat*	Huber et al. (2000); Miura et al. (2000); Koda et al. (2004)
	Astrocytes	Non-injured	*Rat*	Liu et al. (1997), (2010); Hermens and Verhaagen, (1997)
		Contusion	*Rat*	Tai et al. (2003); Liu et al. (2010); Chen et al. (2016)
		Transection	*Rat*	Huber et al. (2000); Miura et al. (2000)
	Oligodendrocytes	Non-injured	*Rat*	Liu et al. (1997), (2010); Hermens and Verhaagen, (1997)
		Contusion	*Rat*	Liu et al. (2010)
		Transection	*Rat*	Huber et al. (2000)
Retrograde delivery	Neurons	Non-injured	*Rat*	Nakajima et al. (2007), (2010)
		Compression	*Rat*	Nakajima et al. (2005), (2007), (2010)
		Transection	*Rat*	Zhou and Shine, (2003)
		Spontaneous chronic mechanical compression	*twy/twy mouse*	Uchida et al. (2008), (2012)
	Astrocytes	Compression	*Rat*	Nakajima et al. (2010)
		Spontaneous chronic mechanical compression	*twy/twy mouse*	Uchida et al. (2008), (2012)
	Oligodendrocytes	Compression	*Rat*	Nakajima et al. (2010)
		Spontaneous chronic mechanical compression	*twy/twy mouse*	Uchida et al. (2008), (2012)

The efficacy of rAdV-mediated gene transfer *ex vivo* has been investigated in various cell lines used to treat SCI (Lin et al., 2016). Transduction efficiency in different cells using for *ex vivo* gene transfer is presented in Table 2. Transplantation of mesenchymal stem cells (MSCs) is the most frequently used treatment approach for SCI, and the positive effects of these cells have been documented (Hofstetter et al., 2002; Matyas et al., 2017). The highly efficient transduction of human MSCs with rAdV5 at multiplicity of infection (MOI) of 50–100 has been repeatedly demonstrated (Koda et al., 2007; Pu et al., 2017; Shi et al., 2019). The efficiency of adenoviral transduction of rat MSCs at an MOI of 300 was 90%, with 95% cell viability after 48 h rAdV transduction did not affect cell proliferation or potency, and transgene expression in cells was maintained for at least 21 days *in vivo* (Deng et al., 2004). In another work, the rat MSCs were transduced with rAdV5 at an MOI of 100 with an efficiency of 20%, which increased to 60% at an MOI of 1,000 without affecting the morphology of cells or their ability to differentiate (Rooney et al., 2008). In contrast, another study reported that peak transduction efficiency (50%) occurred at an MOI of 300, and cytotoxicity was observed if the MOI was over 300 (Zhang et al., 2006).

**TABLE 2:** Transduction efficiency in various cell lines using for *ex vivo* gene transfer after SCI.

Cells	Adenoviral vector	Transduction efficiency	Reference
rat MSCs	rAdV5	80% at an MOI of 50 PFU/cell	Pu et al. (2017)
		80% at an MOI of 100 PFU/cell	Koda et al. (2007)
		90% at an MOI of 300 PFU/cell	Deng et al. (2004)
		60% at an MOI of 1000 PFU/cell	Rooney et al. (2008)
		50% at an MOI of 300 PFU/cell	Zhang et al. (2006)
human MSCs	rAdV5	85% at an MOI of 200 PFU/cell	Marasini et al. (2017)
		<5% at an MOI of 5000 VP/cell	Kuroki et al. (2017)
		5–8% at an MOI of 100 PFU/cell	Park et al. (2010)
	AdV5FRGD	30% at an MOI of 3000 VP/cell	Tsuda et al. (2003)
	AdV5pK7	40% at an MOI of 5000 VP/cell	Kuroki et al. (2017)
	AdV5F35	100% at an MOI of 1000 VP/cell	Olmsted-Davis et al. (2002)
		90% at an MOI of 90 IU/cell	Knaän-Shanzer et al. (2005)
	AdV5F50	90% at an MOI of 90 IU/cell	Knaän-Shanzer et al. (2005)
rat SCs	rAdV5	100% at an MOI of 250 PFU/cell	Shy et al. (1995)
		90–95%	Guo et al. (2007)
		90–95% at an MOI of 300 PFU/cell	Zhang et al. (2007)
mice NSPCs	rAdV5	68% at an MOI of 161 PFU/cell, but altered differentiation was observed	Falk et al. (2002)
		Resistant at an MOI of 1000 PFU/cell	Schmidt et al. (2005)
human NSPCs	AdV5pk7 (conditionally replicating adenovirus)	the more effective than AdV5	Tyler et al. (2009)
rat OECs	rAdV5	100% at an MOI of 100 PFU/cell	Ruitenberg et al. (2002), (2003)

MOI, multiplicity of infection; PFU, plaque forming unit; IU, infection unit; VP, viral particle.

Despite the fact that many studies have demonstrated the highly efficient transduction of human and rat MSCs with vectors based on adenovirus serotype 5 (Deng et al., 2004; Kawamura et al., 2005; Li et al., 2006; Zhang et al., 2006; Klöpper et al., 2008; Xu et al., 2009; Marasini et al., 2017; Pu et al., 2017; Shi et al., 2019), there have also been reports showing the opposite results (Park et al., 2010; Kuroki et al., 2017). The low transduction efficiency was attributed to the low expression of coxsackievirus–adenovirus receptor (CAR), a molecule that enables adenovirus serotype 5 to bind to the host cell surface. For human MSCs, it was demonstrated that CAR is expressed in only a small fraction of cells, while there is abundant expression of αvβ3 and αvβ5 integrins (Conget and Minguell, 2000), which play a role in the endocytosis of Ad5 (Lyle and McCormick, 2010). Although AdV5-based vectors have a high potential to transduce a wide range of cells, there are approaches to change the tropism of AdV5 and increase its transduction efficiency by capsid modifications. rAdVs with modified tropism were investigated as resources to increase the efficiency of gene transfer in MSCs. A modified rAdV with an RGD motif–containing peptide inserted in the HI loop of the knob domain of the Ad5 fiber (rAd5F/RGD) was studied. rAd5F/RGD may exploit the integrin complexes αvβ3 and αvβ5 in addition to CAR as its primary receptors. rAd5F/RGD was 12 times more efficient than rAdV5 with the wild-type fiber in transducing MSCs (Tsuda et al., 2003). The fiber chimeric rAdVs Ad5F50, Ad5F35, and Ad5F16, with fiber domains derived from adenoviruses of serotypes 50, 35, and 16, respectively, were more efficient than rAdV5 with wild-type fiber (Olmsted-Davis et al., 2002; Knaän-Shanzer et al., 2005). However, the high efficiency of transduction of human MSCs by these chimeric rAdVs has not been explained. It is known that group B adenoviruses, including adenoviruses of serotypes 50, 35 and 16, interact with the CD46 receptor as their primary receptor (Pearse et al., 2004), which was shown to be expressed at a low level on human MSCs (Knaän-Shanzer et al., 2005).

Schwann cells (SCs) also showed good results in SCI therapy. Many experiments using rAdV5 as a vehicle for gene transfer have demonstrated the high efficiency of rat SC transduction (Shy et al., 1995; Dijkhuizen et al., 1997; Guénard et al., 1999; Watanabe et al., 2006; Guo et al., 2007; Zhang et al., 2007). rAdV5 transduced SCs with almost 100% efficiency at an MOI of 250–300 (Shy et al., 1995; Guo et al., 2007; Zhang et al., 2007). At these MOIs, cytotoxicity was not observed, cells retained their native morphology, and transgene expression *in vitro* was observed for at least 2 weeks with no decrease in the percentage of infected cells (Shy et al., 1995). In another study, rAdV5 infected only 30% of SCs (Golden et al., 2007). To increase the efficiency of SC transduction, one study modified the fiber of rAdV5 by adding a peptide analog of transferrin to the C-terminus of the protein, which allowed rAdV to penetrate into cells through the transferrin receptor. This significantly improved the transduction and expression of a transgene in neuroglial cells, including SCs (Joung et al., 2005).

Another commonly used cell type is neural stem/progenitor cells (NSPCs) (Lu et al., 2003; Karimi-Abdolrezaee et al., 2006; Ziv et al., 2006; Hwang et al., 2019). It was reported that the efficiency of NSPC transduction by rAdV5 was 68%, but spontaneous differentiation was observed in some cases (Falk et al., 2002). However, in another study, infection with rAdVs did not affect the morphology, survival, proliferation, or differentiation of NSPCs (Hwang et al., 2019). There is also conflicting evidence that NSPCs have a wide range of receptors for various adenoviruses, such as CAR, αvβ3, αvβ5, CD46 and syndecan. For example, NSPCs were shown to lack CAR and αvβ5 on the surface, resulting in their complete resistance to rAdV5 transduction even at an MOI of 1,000 (Schmidt et al., 2005). In contrast, it was also reported that the most effective vectors were those based on the AdV5 serotype and a vector AdV5pk7 containing a poly-L-lysine (pk7) peptide in the fiber knob domain (Tyler et al., 2009).

Olfactory ensheathing cells (OECs) have also demonstrated success in the therapy of experimental SCI (Mayeur et al., 2013; Stepanova et al., 2019). OECs were susceptible to infection with rAdV5, reaching 100% transduction efficiency at an MOI of 100 without observable changes in their cellular phenotype or expression of cell markers. Transgene expression was observed for approximately 30 days after OEC transplantation into the damaged spinal cord (Ruitenberg et al., 2002, 2003).

Thus, adenovectors based on AdV5 and fiber-modified derivatives may have relatively high efficiency in transducing the cell types conventionally used for SCI therapy.

## The Immune Response to Recombinant Adenoviruses in the Context of SCI Therapy

The ability of viral vectors to activate the immune system is a serious limitation the use of such delivery systems, particularly rAdVs. One possible outcome of immune activation is the rapid plateauing of the expression of a therapeutic transgene. In addition, immune activation can lead to damage to healthy cells and the development of unwanted inflammation. This is especially true for SCI therapy, since inflammation may lead to disruption of the regenerative processes. Replication-incompetent rAdVs are capable of causing acute inflammation in infected tissues by activating the innate immune system and stimulating the expression of multiple chemokines and cytokines in transduced target cells (Zaiss et al., 2002; Muruve, 2004; Ghosh et al., 2006). As noted above, the use of second- and third-generation rAdVs avoids the negative impact of immune activation. Since most studies of rAdVs have explored only the first generation of vectors, an important consideration in applying rAdVs is to understand the mechanisms of the immune system response.

When AdV5-LacZ was directly injected into the injured spinal cord of rat, an early immune response was observed at the injection site. One week later, a dense accumulation of OX-42-positive cells (macrophages, lymphocytes, microglia) was observed at the injection site, and some of these cells expressed the transgene (Liu et al., 1997). β-Galactosidase-positive cells were surrounded by a large number of activated microglia. After 1 month, most of the cells expressing the transgene disappeared, and only a few cells along the needle track surrounded by the zone of dense immunoreactivity were weakly stained. The immune response ceased when the β-galactosidase-positive cells disappeared after 2 months. In contrast, no immune response was observed at the site of distant expression of the transgene, even though transgene expression continued throughout the observation period (2 months). No strong immune response and no significant decrease in the number of β-galactosidase-positive cells at the site of virus injection were observed in animals treated with cyclosporin A (Liu et al., 1997). Regarding the immune response evoked by the direct injection of an rAdV or lentiviral vector, an intense immune response was observed only after rAdV injection, and this response could be blocked the intraperitoneal injection of monoclonal antibodies against lymphocytic receptors CD4 and CD45 (Abdellatif et al., 2006). Infiltration of CD8^+^ lymphocytes was noted at the injection site of rAdV5 at an MOI of 5 × 10^6^ pfu, while at the low doses, there were no differences in comparison with the control rats regarding the level of the inflammatory response associated with the presence of macrophages, reactive astrocytes, and microglia (Hermens and Verhaagen, 1997; Huber et al., 2000).

Immunohistological analysis of the damaged spinal cord after a retrograde injection of an rAdV did not reveal any differences in the number of macrophages compared to the control animals, suggesting that retrograde rAdV delivery did not cause widespread inflammation (Zhou et al., 2003). In addition, the time of transgene expression was increased after a retrograde injection compared to a direct injection, indicating a lower immune response (Baumgartner and Shine, 1998). In contrast, there is data indicating equal expression time of transgenes after direct and retrograde injections, in association with equal numbers of axons (Zhou and Shine, 2003). Thus, it is possible that the inflammation around neuronal cell bodies in association with direct rAdV5 injection neither inhibited nor amplified axonal regrowth (Zhou and Shine, 2003).

Host immune response directed against the transgene product and/or virus proteins triggers clearance of the transplanted cells after *ex vivo* gene therapy. A significant reduction in the survival rate of transduced cells after transplantation can indirectly indicate the activation of the immune response. MSCs transplanted into the damaged spinal cord may maintain viability for 4–8 weeks (Hofstetter et al., 2002; Jung et al., 2009; Hu et al., 2010; Pal et al., 2010; Torres-Espín et al., 2015; Hakim et al., 2019). The average survival time of MSCs transduced with various rAdVs is 4–5 weeks (Sasaki et al., 2009; Mukhamedshina et al., 2016; Islamov et al., 2017a; Hei et al., 2017; Shi et al., 2019). Additionally, the expression of an EGFP transgene by MSCs was detected on the 14th day (Mukhamedshina et al., 2016; Islamov et al., 2017b), while only individual EGFP-positive cells were found on the 21st day (Mukhamedshina et al., 2016). Similarly, the expression of a neurotrophin 3 (NT-3) transgene in MSCs was observed for only 14 days, while transplanted cells were detected even on the 67th day (Zhang et al., 2010).

SCs implanted into the spinal cord after contusion damage showed a very long survival time of up to 24 weeks after injection, and the implanted cells were able to proliferate within 12 weeks after transplantation (Wang and Xu, 2014). Similar results were found by other authors, who reported that SCs were detected for 3 months after transplantation (Biernaskie et al., 2007; Pearse et al., 2007; Patel et al., 2010). The transduced SCs transplanted into the damaged spinal cord survived for at least 2 months (Golden et al., 2007; Guo et al., 2007; Zhang et al., 2007). Moreover, the expression of the LacZ transgene was sustained throughout the entire period of SC survival (Zhang et al., 2007). On the other hand, it was reported that the expression of the EGFP transgene was decreased (Golden et al., 2007).

Long-term cell survival (8–11 weeks) was also observed upon NSPC implantation (Ziv et al., 2006; Biernaskie et al., 2007; Hwang et al., 2019), and transduction of NSPCs by rAdVs did not change the duration of cell survival after implantation (Biernaskie et al., 2007; Hwang et al., 2019). Long-term survival (spanning several months) has also been shown for OECs used for SCI therapy (Ruitenberg et al., 2002, 2003; Mayeur et al., 2013; Stepanova et al., 2019). rAdV-mediated expression of a transgene also did not decrease OEC survival after implantation, but transgene expression gradually decreased between the 7th and 30th days after implantation (Ruitenberg et al., 2002).

Thus, the immune response seems to be a minor obstacle to the use of rAdVs for gene transfer in SCI therapy with indirect methods of injection, such as the retrograde administration of rAdVs and the implantation of transduced cells. However, none of the studies discussed above determined the role of preexisting immunity to rAdVs in determining the therapeutic effect of gene therapy, although it is known that neutralizing antibodies against AdV5 are present in 60–90% of the human population (Nwanegbo et al., 2004; Yu et al., 2012).

## The Use of Recombinant Adenoviruses Equipped With Neurotrophic Factors for SCI Therapy

A large amount of research in the field of SCI therapy is focused on using various neurotrophic factors that regulate the survival of damaged neurons, the regeneration of axons, synaptic plasticity, and neurotransmission (Boyce and Mendell, 2014). NT-3, BDNF, and GDNF are the major neurotrophic factors. Neurotrophic factors contribute to the survival of damaged cells and the regeneration of axons (Boyce and Mendell, 2014). Many studies have shown that gene delivery of neurotrophins during SCI therapy may contribute to morphological (Zhou and Shine, 2003; Koda et al., 2004; Nakajima et al., 2005, 2007; Uchida et al., 2008; Zhao et al., 2012; Wang and Xu, 2014) and functional spinal cord recovery (Koda et al., 2004; Zhao et al., 2012; Wang and Xu, 2014; Mukhamedshina et al., 2016; Nejati et al., 2020) after injury.

The retrograde administration of rAdV5-NT-3 to rats with a dissected corticospinal tract led to the growth of axons (Zhou and Shine, 2003). An increase in the axon length and branching of neurons was observed with the retrograde administration of rAdV5-NT-3 to mice with spontaneous chronic mechanical compression of the spinal cord (Uchida et al., 2008). The implantation of retinoic acid–treated MSCs producing NT-3 into the severed spinal cord of rats resulted in a reduced volume of cystic cavities, stimulation of axon regeneration, increased neuron survival, and improved motor function of the hind limbs (Wang and Xu, 2014). Cotransplantation of SC-NT-3 and NSPC expressing the TrkC receptor for NT-3 increased the level of neuronal differentiation of NSCs, stimulated synaptogenesis, induced the formation of myelin SCs, promoted neuroprotection and proliferation of serotonergic fibers in the affected spinal cord, led to a decrease in the main inhibitors of axon growth and neuroplasticity, and resulted in a significant improvement in the retransmission of cortical evoked motor responses and cortical somatosensory evoked potentials as well as a reduction in hindlimb deficits (Wang X. et al., 2011).

The direct injection of rAdV5 expressing BDNF into the dissected spinal cord of rats caused regeneration of descending rubrospinal axons and significant restoration of hindlimb locomotor function (Koda et al., 2004). The retrograde administration of rAdV-BDNF to rats with compression-induced SCI resulted in suppression of neuronal death (Nakajima et al., 2005, 2007; Zhao et al., 2012) and oligodendrocyte death (Nakajima et al., 2005) as well as improvement of behavioral function (Zhao et al., 2012). Similar results were obtained in genetically modified mice with chronic spinal cord compression (Uchida et al., 2012).

Improved recovery of the musculoskeletal system was found in rats with a dissected corticospinal tract that received injections of MSC-BDNF, which promoted axon germination and cortical neuron survival (Sasaki et al., 2009). The implantation of a graft containing Matrigel with rAdV-BDNF-infected MSCs into a completely severed spinal cord resulted in axon regeneration (Koda et al., 2007). The implantation of MSCs-BDNF into rats with a damaged sciatic nerve also led to regeneration of peripheral nerves and improved functional parameters (Hei et al., 2017). The treatment of cervical SCI in rats using OECs infected with rAdV-BDNF reduced the lesion volume, enhanced the regenerative growth of the rubrospinal tract, and improved the recovery of hindlimb function (Ruitenberg et al., 2003).

The direct injection of rAdV-GDNF into the spinal cord after concussion injury or electrolytic damage preserved neuronal fibers and contributed to the restoration of motor function in the hindlimbs (Tai et al., 2003; Tang et al., 2004). Both direct administration of rAdV-GDNF and implantation of MSCs infected with rAdV-GDNF resulted in improved motor function in rats with SCI (Mukhamedshina et al., 2016). The implantation of rAdV-GDNF-transduced NSPCs after spinal cord contusion significantly reduced the volume of lesions and the formation of glial scars, promoted the regeneration of axons and of neurites in general, increased myelination due to increased SC migration, and led to improved recovery of the musculoskeletal system (Hwang et al., 2019). The implantation of a polycaprolactone/collagen scaffold loaded with emu oil and seeded with adipose tissue–induced MSCs infected with rAdV-GDNF at the site of spinal cord injury in rats decreased lesion cavity size and axon demyelination, and a significant recovery of motor function on the was recorded BBB scale (Nejati et al., 2020).

## Conclusion

The natural tropism of rAdV5-based vectors allows transduction of both nerve and glial cells, providing a high level of transgene expression. In addition, rAdV5-based vectors with modified tropism may infect various cells used for SCI therapy. AdV5 is considered to have significant immunogenicity, which can place limits on its successful use. Although this problem is less substantial for second- and especially third-generation AdVs, nevertheless, research has focused mostly on the therapeutic potential of the first-generation rAdVs. The immunogenicity of first-generation rAdVs significantly limits direct administration of these rAdVs to the injury site. In contrast, retrograde administration of rAdVs and implantation of rAdV-transduced cells may not provoke an immune response. However, due to high seropositivity rates for rAdV5 in the human population, further research on animals with preexisting anti-AdV5 antibodies is necessary to confirm this immune-sparing effect. Moreover, it is unclear how preexisting antibodies will affect the efficacy of adenoviral SCI therapy due to the presence of a blood-cerebrospinal fluid barrier. It was shown that preimmunization of animals with AdV5 prior to the delivery of first-generation rAdVs into the brain significantly reduced the expression of a transgene while not affecting “gutless” rAdVs (Barcia et al., 2007). The third generation rADVs is widely studied for gene delivery to the central nervous system after some neurological disease (Ricobaraz et al., 2020), but we could not find published data on using the third generation rADVs for SCI treatment. It is also notable that first-generation rAdVs provide relatively short periods of transgene expression; these expression periods may be sufficient to achieve a positive therapeutic effect in SCI models, but it remains unclear whether such short-term expression of therapeutic genes will be sufficient for SCI therapy in humans. Based on the available preclinical data, SCI therapies using rAdVs are promising and should be investigated further.

## References

[B1] Abdellatif A. A. Pelt J. L. Benton R. L. Howard R. M. Tsoulfas P. Ping P. (2006). Gene Delivery to the Spinal Cord: Comparison between Lentiviral, Adenoviral, and Retroviral Vector Delivery Systems. J. Neurosci. Res. 84 (3), 553–567. 10.1002/jnr.20968 16786574 PMC2862356

[B2] Alba R. Bosch A. Chillon M. (2005). Gutless Adenovirus: Last-Generation Adenovirus for Gene Therapy. Gene Ther. 12 (1), S18–S27. 10.1038/sj.gt.3302612 16231052

[B3] Barcia C. Jimenez-Dalmaroni M. Kroeger K. M. Puntel M. Rapaport A. J. Larocque D. (2007). One-year Expression from High-Capacity Adenoviral Vectors in the Brains of Animals with Pre-existing Anti-adenoviral Immunity: Clinical Implications. Mol. Ther. 15 (12), 2154–2163. 10.1038/sj.mt.6300305 17895861 PMC2268647

[B4] Baumgartner B. J. Shine H. D. (1998). Neuroprotection of Spinal Motoneurons Following Targeted Transduction with an Adenoviral Vector Carrying the Gene for Glial Cell Line-Derived Neurotrophic Factor. Exp. Neurol. 153 (1), 102–112. 10.1006/exnr.1998.6878 9743571

[B5] Betz V. M. Sitoci-Ficici K. H. Uckermann O. Leipnitz E. Iltzsche A. Thirion C. (2016). Gene-activated Fat Grafts for the Repair of Spinal Cord Injury: A Pilot Study. Acta neurochirurgica 158 (2), 367–378. 10.1007/s00701-015-2626-y 26592254

[B6] Biernaskie J. Sparling J. S. Liu J. Shannon C. P. Plemel J. R. Xie Y. (2007). Skin-derived Precursors Generate Myelinating Schwann Cells that Promote Remyelination and Functional Recovery after Contusion Spinal Cord Injury. J. Neurosci. 27 (36), 9545–9559. 10.1523/JNEUROSCI.1930-07.2007 17804616 PMC6672973

[B7] Blesch A. Tuszynski M. H. (2007). Transient Growth Factor Delivery Sustains Regenerated Axons after Spinal Cord Injury. J. Neurosci. 27 (39), 10535–10545. 10.1523/JNEUROSCI.1903-07.2007 17898225 PMC6673161

[B8] Blits B. Bunge M. B. (2006). Direct Gene Therapy for Repair of the Spinal Cord. J. neurotrauma 23 (3-4), 508–520. 10.1089/neu.2006.23.508 16629633

[B9] Blits B. Dijkhuizen P. A. Hermens W. T. Van Esseveldt L. K. Boer G. J. Verhaagen J. (2000). The Use of Adenoviral Vectors and *Ex Vivo* Transduced Neurotransplants: towards Promotion of Neuroregeneration. Cel Transplant. 9 (2), 169–178. 10.1177/096368970000900204 10811391

[B10] Boyce V. S. Mendell L. M. (2014). ““Neurotrophic Factors in Spinal Cord Injury” in Neurotrophic Factors,” in Handbook of Experimental Pharmacology. Editors Lewin G. Carter B. (Berlin, Heidelberg: Springer), 220. 10.1007/978-3-642-45106-5_16 24668482

[B11] Brunetti-Pierri N. Stapleton G. E. Law M. Breinholt J. Palmer D. J. Zuo Y. (2009). Efficient, Long-Term Hepatic Gene Transfer Using Clinically Relevant HDAd Doses by Balloon Occlusion Catheter Delivery in Nonhuman Primates. Mol. Ther. 17 (2), 327–333. 10.1038/mt.2008.257 19050700 PMC2835071

[B12] Bydon M. Lin J. Macki M. Gokaslan Z. L. Bydon A. (2014). The Current Role of Steroids in Acute Spinal Cord Injury. World Neurosurg. 82 (5), 848–854. 10.1016/j.wneu.2013.02.062 23454689

[B13] Chen C. H. Sung C. S. Huang S. Y. Feng C. W. Hung H. C. Yang S. N. (2016). The Role of the PI3K/Akt/mTOR Pathway in Glial Scar Formation Following Spinal Cord Injury. Exp. Neurol. 278, 27–41. 10.1016/j.expneurol.2016.01.023 26828688

[B14] Conget P. A. Minguell J. J. (2000). Adenoviral-mediated Gene Transfer into *Ex Vivo* Expanded Human Bone Marrow Mesenchymal Progenitor Cells. Exp. Hematol. 28 (4), 382–390. 10.1016/S0301-472X(00)00134-X 10781896

[B15] Crystal R. G. (2014). Adenovirus: the First Effective *In Vivo* Gene Delivery Vector. Hum. Gene Ther. 25 (1), 3–11. 10.1089/hum.2013.2527 24444179 PMC3900005

[B16] Danthinne X. Imperiale M. J. (2000). Production of First Generation Adenovirus Vectors: a Review. Gene Ther. 7 (20), 1707–1714. 10.1038/sj.gt.3301301 11083491

[B17] Deng W. Bivalacqua T. J. Chattergoon N. N. Jeter J. R. Jr Kadowitz P. J. (2004). Engineering Ex Vivo–Expanded Marrow Stromal Cells to Secrete Calcitonin Gene–Related Peptide Using Adenoviral Vector. Stem Cells 22 (7), 1279–1291. 10.1634/stemcells.2004-0032 15579646

[B18] Dijkhuizen P. A. Hermens W. T. Teunis M. A. Verhaagen J. (1997). Adenoviral Vector‐directed Expression of Neurotrophin‐3 in Rat Dorsal Root Ganglion Explants Results in a Robust Neurite Outgrowth Response. J. Neurobiol. 33 (2), 172–184. 10.1002/(SICI)1097-4695(199708)33:2<172:AID-NEU6>3.0.CO;2-# 9240373

[B19] Falk A. Holmström N. Carlén M. Cassidy R. Lundberg C. Frisén J. (2002). Gene Delivery to Adult Neural Stem Cells. Exp. Cel. Res. 279 (1), 34–39. 10.1006/excr.2002.5569 12213211

[B20] Fouad K. Bennett D. Vavrek R. Blesch A. (2013). Long-term Viral Brain-Derived Neurotrophic Factor Delivery Promotes Spasticity in Rats with a Cervical Spinal Cord Hemisection. Front. Neurol. 4, 187. 10.3389/fneur.2013.00187 24312075 PMC3832889

[B21] Ghosh S. S. Gopinath P. Ramesh A. (2006). Adenoviral Vectors. Appl. Biochem. Biotechnol. 133 (1), 9–29. 10.1385/ABAB:133:1:9 16622281

[B22] Golden K. L. Pearse D. D. Blits B. Garg M. S. Oudega M. Wood P. M. (2007). Transduced Schwann Cells Promote Axon Growth and Myelination after Spinal Cord Injury. Exp. Neurol. 207 (2), 203–217. 10.1016/j.expneurol.2007.06.023 17719577 PMC3513343

[B23] Guénard V. Schweitzer B. Flechsig E. Hemmi S. Martini R. Suter U. (1999). Effective Gene Transfer of lacZ and P0 into Schwann Cells of P0-Deficient Mice. Glia 25 (2), 165–178. 10.1002/(SICI)1098-1136(19990115)25:2<165:AID-GLIA7>3.0.CO;2-L 9890631

[B24] Guo J. S. Zeng Y. S. Li H. B. Huang W. L. Liu R. Y. Li X. B. (2007). Cotransplant of Neural Stem Cells and NT-3 Gene Modified Schwann Cells Promote the Recovery of Transected Spinal Cord Injury. Spinal cord 45 (1), 15–24. 10.1038/sj.sc.3101943 16773039

[B25] Hakim R. Covacu R. Zachariadis V. Frostell A. Sankavaram S. R. Brundin L. (2019). Mesenchymal Stem Cells Transplanted into Spinal Cord Injury Adopt Immune Cell-like Characteristics. Stem Cel. Res. Ther. 10 (1), 1–16. 10.1186/s13287-019-1218-9 PMC6448247 30944028

[B26] Hei W. H. Almansoori A. A. Sung M. A. Ju K. W. Seo N. Lee S. H. (2017). Adenovirus Vector-Mediated *Ex Vivo* Gene Transfer of Brain-Derived Neurotrophic Factor (BDNF) Tohuman Umbilical Cord Blood-Derived Mesenchymal Stem Cells (UCB-MSCs) Promotescrush-Injured Rat Sciatic Nerve Regeneration. Neurosci. Lett. 643, 111–120. 10.1016/j.neulet.2017.02.030 28215880

[B27] Hendriks W. T. Ruitenberg M. J. Blits B. Boer G. J. Verhaagen J. (2004). Viral Vector-Mediated Gene Transfer of Neurotrophins to Promote Regeneration of the Injured Spinal Cord. Prog. Brain Res. 146, 451–476. 10.1016/S0079-6123(03)46029-9 14699980

[B28] Hermens W. T. Verhaagen J. (1997). Adenoviral Vector-Mediated Gene Expression in the Nervous System of Immunocompetent Wistar and T Cell-Deficient Nude Rats: Preferential Survival of Transduced Astroglial Cells in Nude Rats. Hum. Gene Ther. 8 (9), 1049–1063. 10.1089/hum.1997.8.9-1049 9189763

[B29] Hermens W. T. Verhaagen J. (1998). Suppression of Inflammation by Dexamethasone Prolongs Adenoviral Vector-Mediated Transgene Expression in the Facial Nucleus of the Rat. Brain Res. Bull. 47 (2), 133–140. 10.1016/S0361-9230(98)00042-2 9820730

[B30] Hofstetter C. P. Schwarz E. J. Hess D. Widenfalk J. El Manira A. Prockop D. J. (2002). Marrow Stromal Cells Form Guiding Strands in the Injured Spinal Cord and Promote Recovery. Proc. Natl. Acad. Sci. 99 (4), 2199–2204. 10.1073/pnas.042678299 11854516 PMC122342

[B31] Horch H. W. (2004). Local Effects of BDNF on Dendritic Growth. Rev. neurosciences 15 (2), 117–130. 10.1515/REVNEURO.2004.15.2.117 15202684

[B32] Hu S. L. Luo H. S. Li J. T. Xia Y. Z. Li L. Zhang L. J. (2010). Functional Recovery in Acute Traumatic Spinal Cord Injury after Transplantation of Human Umbilical Cord Mesenchymal Stem Cells. Crit. Care Med. 38 (11), 2181–2189. 10.1097/CCM.0b013e3181f17c0e 20711072

[B33] Huber A. B. Ehrengruber M. U. Schwab M. E. Brösamle C. (2000). Adenoviral Gene Transfer to the Injured Spinal Cord of the Adult Rat. Eur. J. Neurosci. 12 (9), 3437–3442. 10.1046/j.1460-9568.2000.00255.x 10998127

[B34] Hwang K. Jung K. Kim I. S. Kim M. Han J. Lim J. (2019). Glial Cell Line-Derived Neurotrophic Factor-Overexpressing Human Neural Stem/progenitor Cells Enhance Therapeutic Efficiency in Rat with Traumatic Spinal Cord Injury. Exp. Neurobiol. 28 (6), 679. 10.5607/en.2019.28.6.679 31902156 PMC6946112

[B35] Islamov R. R. Izmailov A. A. Sokolov M. E. Fadeev P. O. Bashirov F. V. Eremeev A. A. (2017a). Evaluation of Direct and Cell-Mediated Triple-Gene Therapy in Spinal Cord Injury in Rats. Brain Res. Bull. 132, 44–52. 10.1016/j.brainresbull.2017.05.005 28529158

[B36] Islamov R. R. Sokolov M. E. Bashirov F. V. Fadeev F. O. Shmarov M. M. Naroditskiy B. S. (2017b). A Pilot Study of Cell-Mediated Gene Therapy for Spinal Cord Injury in Mini Pigs. Neurosci. Lett. 644, 67–75. 10.1016/j.neulet.2017.02.034 28213069

[B37] Jones L. L. Oudega M. Bunge M. B. Tuszynski M. H. (2001). Neurotrophic Factors, Cellular Bridges and Gene Therapy for Spinal Cord Injury. J. Physiol. 533 (1), 83–89. 10.1111/j.1469-7793.2001.0083b.x 11351016 PMC2278599

[B38] Joung I. Harber G. Gerecke K. M. Carroll S. L. Collawn J. F. Engler J. A. (2005). Improved Gene Delivery into Neuroglial Cells Using a Fiber-Modified Adenovirus Vector. Biochem. biophysical Res. Commun. 328 (4), 1182–1187. 10.1016/j.bbrc.2005.01.080 15708001

[B39] Jung D. I. Ha J. Kang B. T. Kim J. W. Quan F. S. Lee J. H. (2009). A Comparison of Autologous and Allogenic Bone Marrow-Derived Mesenchymal Stem Cell Transplantation in Canine Spinal Cord Injury. J. Neurol. Sci. 285 (1-2), 67–77. 10.1016/j.jns.2009.05.027 19555980

[B40] Karimi-Abdolrezaee S. Eftekharpour E. Wang J. Morshead C. M. Fehlings M. G. (2006). Delayed Transplantation of Adult Neural Precursor Cells Promotes Remyelination and Functional Neurological Recovery after Spinal Cord Injury. J. Neurosci. 26 (13), 3377–3389. 10.1523/JNEUROSCI.4184-05.2006 16571744 PMC6673854

[B41] Kawamura K. Chu C. R. Sobajima S. Robbins P. D. Fu F. H. Izzo N. J. (2005). Adenoviral-mediated Transfer of TGF-Β1 but Not IGF-1 Induces Chondrogenic Differentiation of Human Mesenchymal Stem Cells in Pellet Cultures. Exp. Hematol. 33 (8), 865–872. 10.1016/j.exphem.2005.05.010 16038778 PMC1360180

[B42] Klöpper J. Lindenmaier W. Fiedler U. Mehlhorn A. Stark G. B. Finkenzeller G. (2008). High Efficient Adenoviral-Mediated VEGF and Ang-1 Gene Delivery into Osteogenically Differentiated Human Mesenchymal Stem Cells. Microvasc. Res. 75 (1), 83–90. 10.1016/j.mvr.2007.04.010 17603084

[B43] Knaän‐Shanzer S. van de Watering M. J. van der Velde I. Gonçalves M. A. Valerio D. de Vries A. A. (2005). Endowing Human Adenovirus Serotype 5 Vectors with Fiber Domains of Species B Greatly Enhances Gene Transfer into Human Mesenchymal Stem Cells. Stem Cells 23 (10), 1598–1607. 10.1634/stemcells.2005-0016 16293583

[B44] Koda M. Hashimoto M. Murakami M. Yoshinaga K. Ikeda O. Yamazaki M. (2004). Adenovirus Vector-Mediated *In Vivo* Gene Transfer of Brain-Derived Neurotrophic Factor (BDNF) Promotes Rubrospinal Axonal Regeneration and Functional Recovery after Complete Transection of the Adult Rat Spinal Cord. J. neurotrauma 21 (3), 329–337. 10.1089/089771504322972112 15115607

[B45] Koda M. Kamada T. Hashimoto M. Murakami M. Shirasawa H. Sakao S. (2007). Adenovirus Vector-Mediated *Ex Vivo* Gene Transfer of Brain-Derived Neurotrophic Factor to Bone Marrow Stromal Cells Promotes Axonal Regeneration after Transplantation in Completely Transected Adult Rat Spinal Cord. Eur. Spine J. 16 (12), 2206–2214. 10.1007/s00586-007-0499-3 17885772 PMC2140138

[B46] Kuipers S. D. Bramham C. R. (2006). Brain-derived Neurotrophic Factor Mechanisms and Function in Adult Synaptic Plasticity: New Insights and Implications for Therapy. Curr. Opin. Drug Discov. Devel 9 (5), 580–586. 10.1016/j.pneurobio.2005.06.003 17002218

[B47] Kuroki L. M. Jin X. Dmitriev I. P. Kashentseva E. A. Powell M. A. Mutch D. G. (2017). Adenovirus Platform Enhances Transduction Efficiency of Human Mesenchymal Stem Cells: An Opportunity for Cellular Carriers of Targeted TRAIL-Based TR3 Biologics in Ovarian Cancer. PloS one 12 (12), e0190125. 10.1371/journal.pone.0190125 29267342 PMC5739501

[B48] Lee C. S. Bishop E. S. Zhang R. Yu X. Farina E. M. Yan S. (2017). Adenovirus-mediated Gene Delivery: Potential Applications for Gene and Cell-Based Therapies in the new era of Personalized Medicine. Genes Dis. 4 (2), 43–63. 10.1016/j.gendis.2017.04.001 28944281 PMC5609467

[B49] Li X. Lu Y. Huang W. Xu H. Chen X. Geng Q. (2006). *In Vitro* effect of Adenovirus‐mediated Human Gamma Interferon Gene Transfer into Human Mesenchymal Stem Cells for Chronic Myelogenous Leukemia. Hematological Oncol. 24 (3), 151–158. 10.1002/hon.779 16700092

[B50] Lin X. Y. Lai B. Q. Zeng X. Che M. T. Ling E. A. Wu W. (2016). Cell Transplantation and Neuroengineering Approach for Spinal Cord Injury Treatment: a Summary of Current Laboratory Findings and Review of Literature. Cel Transplant. 25 (8), 1425–1438. 10.3727/096368916X690836 26850705

[B51] Liu Y. Figley S. Spratt S. K. Lee G. Ando D. Surosky R. (2010). An Engineered Transcription Factor Which Activates VEGF-A Enhances Recovery after Spinal Cord Injury. Neurobiol. Dis. 37 (2), 384–393. 10.1016/j.nbd.2009.10.018 19879362

[B52] Liu Y. Himes B. T. Moul J. Huang W. Chow S. Y. Tessler A. (1997). Application of Recombinant Adenovirus for *In Vivo* Gene Delivery to Spinal Cord. Brain Res. 768 (1-2), 19–29. 10.1016/S0006-8993(97)00587-8 9369296

[B53] Lu P. Jones L. L. Snyder E. Y. Tuszynski M. H. (2003). Neural Stem Cells Constitutively Secrete Neurotrophic Factors and Promote Extensive Host Axonal Growth after Spinal Cord Injury. Exp. Neurol. 181 (2), 115–129. 10.1016/S0014-4886(03)00037-2 12781986

[B54] Lyle C. McCormick F. (2010). Integrin αvβ5 Is a Primary Receptor for Adenovirus in CAR-Negative Cells. Virol. J. 7 (1), 1–13. 10.1186/1743-422X-7-148 20615244 PMC2909962

[B55] Maier I. C. Schwab M. E. (2006). Sprouting, Regeneration and Circuit Formation in the Injured Spinal Cord: Factors and Activity. Philos. Trans. R. Soc. B: Biol. Sci. 361 (1473), 1611–1634. 10.1098/rstb.2006.1890 PMC1664674 16939978

[B56] Marasini S. Chang D. Y. Jung J. H. Lee S. J. Cha H. L. Suh-Kim H. (2017). Effects of Adenoviral Gene Transduction on the Stemness of Human Bone Marrow Mesenchymal Stem Cells. Mol. Cell 40 (8), 598. 10.14348/molcells.2017.0095 PMC5582306 28835020

[B57] Matyas J. J. Stewart A. N. Goldsmith A. Nan Z. Skeel R. L. Rossignol J. (2017). Effects of Bone-Marrow–Derived MSC Transplantation on Functional Recovery in a Rat Model of Spinal Cord Injury: Comparisons of Transplant Locations and Cell Concentrations. Cel Transplant. 26 (8), 1472–1482. 10.1177/0963689717721214 PMC5680979 28901182

[B58] Mayeur A. Duclos C. Honore A. Gauberti M. Drouot L. do Rego J. C. (2013). Potential of Olfactory Ensheathing Cells from Different Sources for Spinal Cord Repair. PLoS One 8 (4), e62860. 10.1371/journal.pone.0062860 23638158 PMC3634744

[B59] Miura T. Tanaka S. Seichi A. Arai M. Goto T. Katagiri H. (2000). Partial Functional Recovery of Paraplegic Rat by Adenovirus-Mediated Gene Delivery of Constitutively Active MEK1. Exp. Neurol. 166 (1), 115–126. 10.1006/exnr.2000.7493 11031088

[B60] Mohan R. Tosolini A. P. Morris R. (2015). Intramuscular Injections along the Motor End Plates: a Minimally Invasive Approach to Shuttle Tracers Directly into Motor Neurons. JoVE (Journal of Visualized Experiments) 101, e52846. 10.3791/52846 PMC4545031 26273739

[B61] Mohan R. Tosolini A. P. Morris R. (2014). Targeting the Motor End Plates in the Mouse Hindlimb Gives Access to a Greater Number of Spinal Cord Motor Neurons: an Approach to Maximize Retrograde Transport. Neuroscience 274, 318–330. 10.1016/j.neuroscience.2014.05.045 24892760

[B62] Mukhamedshina Y. O. Shaymardanova G. F. Garanina Е. Е. Salafutdinov I. I. Rizvanov А. А. Islamov R. R. (2016). Adenoviral Vector Carrying Glial Cell-Derived Neurotrophic Factor for Direct Gene Therapy in Comparison with Human Umbilical Cord Blood Cell-Mediated Therapy of Spinal Cord Injury in Rat. Spinal Cord 54 (5), 347–359. 10.1038/sc.2015.161 26415641

[B63] Muruve D. A. (2004). The Innate Immune Response to Adenovirus Vectors. Hum. Gene Ther. 15 (12), 1157–1166. 10.1089/hum.2004.15.1157 15684693

[B64] Nakajima H. Uchida K. Kobayashi S. Inukai T. Horiuchi Y. Yayama T. (2007). Rescue of Rat Anterior Horn Neurons after Spinal Cord Injury by Retrograde Transfection of Adenovirus Vector Carrying Brain-Derived Neurotrophic Factor Gene. J. neurotrauma 24 (4), 703–712. 10.1089/neu.2006.0004 17439352

[B65] Nakajima H. Uchida K. Kobayashi S. Kokubo Y. Yayama T. Sato R. (2005). Targeted Retrograde Gene Delivery into the Injured Cervical Spinal Cord Using Recombinant Adenovirus Vector. Neurosci. Lett. 385 (1), 30–35. 10.1016/j.neulet.2005.05.012 15936879

[B66] Nakajima H. Uchida K. Yayama T. Kobayashi S. Guerrero A. R. Furukawa S. (2010). Targeted Retrograde Gene Delivery of Brain-Derived Neurotrophic Factor Suppresses Apoptosis of Neurons and Oligodendroglia after Spinal Cord Injury in Rats. Spine 35 (5), 497–504. 10.1097/BRS.0b013e3181b8e89b 20190624

[B67] Nejati K. Mehdi D. Ghareghomi S. Mostafavi E. Ebrahimi-Kalan A. Biglari A. (2020). GDNF Gene-Engineered Adipose-Derived Stem Cells Seeded Emu Oil-Loaded Electrospun Nanofibers for Axonal Regeneration Following Spinal Cord Injury. J. Drug Deliv. Sci. Techn. 60, 102095. 10.1016/j.jddst.2020.102095

[B68] Nwanegbo E. Vardas E. Gao W. Whittle H. Sun H. Rowe D. (2004). Prevalence of Neutralizing Antibodies to Adenoviral Serotypes 5 and 35 in the Adult Populations of the Gambia, South Africa, and the United States. Clin. Vaccin. Immunol. 11 (2), 351–357. 10.1128/CDLI.11.2.351-357.2004 PMC371218 15013987

[B69] Olmsted-Davis E. A. Gugala Z. Gannon F. H. Yotnda P. McAlhany R. E. Lindsey R. W. (2002). Use of a Chimeric Adenovirus Vector Enhances BMP2 Production and Bone Formation. Hum. Gene Ther. 13 (11), 1337–1347. 10.1089/104303402760128568 12162816

[B70] Pal R. Gopinath C. Rao N. M. Banerjee P. Krishnamoorthy V. Venkataramana N. K. (2010). Functional Recovery after Transplantation of Bone Marrow-Derived Human Mesenchymal Stromal Cells in a Rat Model of Spinal Cord Injury. Cytotherapy 12 (6), 792–806. 10.3109/14653249.2010.487899 20524772

[B71] Park S. H. Doh J. Park S. I. Lim J. Y. Kim S. M. Youn J. I. (2010). Branched Oligomerization of Cell-Permeable Peptides Markedly Enhances the Transduction Efficiency of Adenovirus into Mesenchymal Stem Cells. Gene Ther. 17 (8), 1052–1061. 10.1038/gt.2010.58 20485381

[B72] Parrish J. Z. Emoto K. Kim M. D. Jan Y. N. (2007). Mechanisms that Regulate Establishment, Maintenance, and Remodeling of Dendritic fields. Annu. Rev. Neurosci. 30, 399–423. 10.1146/annurev.neuro.29.051605.112907 17378766

[B73] Patel V. Joseph G. Patel A. Patel S. Bustin D. Mawson D. (2010). Suspension Matrices for Improved Schwann-Cell Survival after Implantation into the Injured Rat Spinal Cord. J. neurotrauma 27 (5), 789–801. 10.1089/neu.2008.0809 20144012 PMC2943946

[B74] Pearse D. D. Pereira F. C. Marcillo A. E. Bates M. L. Berrocal Y. A. Filbin M. T. (2004). cAMP and Schwann Cells Promote Axonal Growth and Functional Recovery after Spinal Cord Injury. Nat. Med. 10 (6), 610–616. 10.1038/nm1056 15156204

[B75] Pearse D. D. Sanchez A. R. Pereira F. C. Andrade C. M. Puzis R. Pressman Y. (2007). Transplantation of Schwann Cells And/or Olfactory Ensheathing Glia into the Contused Spinal Cord: Survival, Migration, Axon Association, and Functional Recovery. Glia 55 (9), 976–1000. 10.1002/glia.20490 17526000

[B76] Povysheva T. Shmarov M. Logunov D. Naroditsky B. Shulman I. Ogurcov S. (2017). Post–spinal Cord Injury Astrocyte-Mediated Functional Recovery in Rats after Intraspinal Injection of the Recombinant Adenoviral Vectors Ad5-VEGF and Ad5-ANG. J. Neurosurg. SPINE 27 (1), 105–115. 10.3171/2016.9.SPINE15959 28452633

[B77] Pu Y. Meng K. Gu C. Wang L. Zhang X. (2017). Thrombospondin-1 Modified Bone Marrow Mesenchymal Stem Cells (BMSCs) Promote Neurite Outgrowth and Functional Recovery in Rats with Spinal Cord Injury. Oncotarget 8 (56), 96276. 10.18632/oncotarget.22018 29221205 PMC5707099

[B78] Ricobaraz A. Gonzalez-Aparicio M. Mora-Jimenez L. Lumbreras S. Hernandez-Alcoceba R. (2020). High-capacity Adenoviral Vectors: Expanding the Scope of Gene Therapy. Int. J. Mol. Sci. 21 (10), 3643. 10.3390/ijms21103643 PMC7279171 32455640

[B79] Rodger J. Drummond E. S. Hellström M. Robertson D. Harvey A. R. (2012). Long-term Gene Therapy Causes Transgene-specific Changes in the Morphology of Regenerating Retinal Ganglion Cells. PLoS One 7 (2), e31061. 10.1371/journal.pone.0031061 22347429 PMC3275572

[B80] Romero M. I. Rangappa N. Li L. Lightfoot E. Garry M. G. Smith G. M. (2000). Extensive Sprouting of Sensory Afferents and Hyperalgesia Induced by Conditional Expression of Nerve Growth Factor in the Adult Spinal Cord. J. Neurosci. 20 (12), 4435–4445. 10.1523/JNEUROSCI.20-12-04435.2000 10844012 PMC6772437

[B81] Rooney G. E. Moran C. McMahon S. S. Ritter T. Maenz M. Flügel A. (2008). Gene-modified Mesenchymal Stem Cells Express Functionally Active Nerve Growth Factor on an Engineered Poly Lactic Glycolic Acid (PLGA) Substrate. Tissue Eng. A 14 (5), 681–690. 10.1089/tea.2007.0260 18402551

[B82] Ruitenberg M. J. Plant G. W. Christensen C. L. Blits B. Niclou S. P. Harvey A. R. (2002). Viral Vector-Mediated Gene Expression in Olfactory Ensheathing Glia Implants in the Lesioned Rat Spinal Cord. Gene Ther. 9 (2), 135–146. 10.1038/sj.gt.3301626 11857072

[B83] Ruitenberg M. J. Plant G. W. Hamers F. P. Wortel J. Blits B. Dijkhuizen P. A. (2003). *Ex Vivo* adenoviral Vector-Mediated Neurotrophin Gene Transfer to Olfactory Ensheathing Glia: Effects on Rubrospinal Tract Regeneration, Lesion Size, and Functional Recovery after Implantation in the Injured Rat Spinal Cord. J. Neurosci. 23 (18), 7045–7058. 10.1523/JNEUROSCI.23-18-07045.2003 12904465 PMC6740651

[B84] Sasaki M. Radtke C. Tan A. M. Zhao P. Hamada H. Houkin K. (2009). BDNF-hypersecreting Human Mesenchymal Stem Cells Promote Functional Recovery, Axonal Sprouting, and protection of Corticospinal Neurons after Spinal Cord Injury. J. Neurosci. 29 (47), 14932–14941. 10.1523/JNEUROSCI.2769-09.2009 19940189 PMC2825276

[B85] Schmidt A. Böckmann M. Stoll A. Racek T. Pützer B. M. (2005). Analysis of Adenovirus Gene Transfer into Adult Neural Stem Cells. Virus. Res. 114 (1-2), 45–53. 10.1016/j.virusres.2005.05.010 15996786

[B86] Shi W. Que Y. Lv D. Bi S. Xu Z. Wang D. (2019). Overexpression of TG2 Enhances the Differentiation of Ectomesenchymal Stem Cells into Neuron-like Cells and Promotes Functional Recovery in Adult Rats Following Spinal Cord Injury. Mol. Med. Rep. 20 (3), 2763–2773. 10.3892/mmr.2019.10502 31322240 PMC6691247

[B87] Shy M. E. Tani M. Shi Y. J. Whyatt S. A. Chbihi T. Scherer S. S. (1995). An Adenoviral Vector Can Transfer lacZ Expression into Schwann Cells in Culture and in Sciatic Nerve. Ann. Neurol. 38 (3), 429–436. 10.1002/ana.410380313 7668829

[B88] Silver J. Miller J. H. (2004). Regeneration beyond the Glial Scar. Nat. Rev. Neurosci. 5 (2), 146–156. 10.1038/nrn1326 14735117

[B89] St George J. A. (2003). Gene Therapy Progress and Prospects: Adenoviral Vectors. Gene Ther. 10 (14), 1135–1141. 10.1038/sj.gt.3302071 12833122

[B90] Stepanova O. V. Voronova A. D. Chadin A. V. Valikhov M. P. Semkina A. S. Karsuntseva E. K. (2019). Efficiency of Human Olfactory Ensheathing Cell Transplantation into Spinal Cysts to Improve Mobility of the Hind Limbs. Stem Cell Dev. 28 (18), 1253–1263. 10.1089/scd.2019.0092 31310179

[B91] Tai M. H. Cheng H. Wu J. P. Liu Y. L. Lin P. R. Kuo J. S. (2003). Gene Transfer of Glial Cell Line-Derived Neurotrophic Factor Promotes Functional Recovery Following Spinal Cord Contusion. Exp. Neurol. 183 (2), 508–515. 10.1016/S0014-4886(03)00130-4 14552891

[B92] Tang X. Q. Wang Y. Huang Z. H. Han J. S. Wan Y. (2004). Adenovirus-mediated Delivery of GDNF Ameliorates Corticospinal Neuronal Atrophy and Motor Function Deficits in Rats with Spinal Cord Injury. Neuroreport 15 (3), 425–429. 10.1097/00001756-200403010-00009 15094497

[B93] Tator C. H. (2006). Review of Treatment Trials in Human Spinal Cord Injury: Issues, Difficulties, and Recommendations. Neurosurgery 59 (5), 957–982. 10.1227/01.NEU.0000245591.16087.89 17143232

[B94] Toietta G. Mane V. P. Norona W. S. Finegold M. J. Ng P. McDonagh A. F. (2005). Lifelong Elimination of Hyperbilirubinemia in the Gunn Rat with a Single Injection of Helper-dependent Adenoviral Vector. Proc. Natl. Acad. Sci. 102 (11), 3930–3935. 10.1073/pnas.0500930102 15753292 PMC554836

[B95] Torres-Espín A. Redondo-Castro E. Hernandez J. Navarro X. (2015). Immunosuppression of Allogenic Mesenchymal Stem Cells Transplantation after Spinal Cord Injury Improves Graft Survival and Beneficial Outcomes. J. neurotrauma 32 (6), 367–380. 10.1089/neu.2014.3562 25203134 PMC4361361

[B96] Tosolini A. P. Mohan R. Morris R. (2013). Targeting the Full Length of the Motor End Plate Regions in the Mouse Forelimb Increases the Uptake of Fluoro-Gold into Corresponding Spinal Cord Motor Neurons. Front. Neurol. 4, 58. 10.3389/fneur.2013.00058 23730296 PMC3657688

[B97] Tosolini A. P. Morris R. (2012). Spatial Characterization of the Motor Neuron Columns Supplying the Rat Forelimb. Neuroscience 200, 19–30. 10.1016/j.neuroscience.2011.10.054 22100785

[B98] Tosolini A. P. Morris R. (2016). Viral-mediated Gene Therapy for Spinal Cord Injury (SCI) from a Translational Neuroanatomical Perspective. Neural Regen. Res. 11 (5), 743–744. 10.4103/1673-5374.182698 27335556 PMC4904463

[B99] Tsintou M. Dalamagkas K. Seifalian A. M. (2015). Advances in Regenerative Therapies for Spinal Cord Injury: a Biomaterials Approach. Neural Regen. Res. 10 (5), 726. 10.4103/1673-5374.156966 26109946 PMC4468763

[B100] Tsuda H. Wada T. Ito Y. Uchida H. Dehari H. Nakamura K. (2003). Efficient BMP2 Gene Transfer and Bone Formation of Mesenchymal Stem Cells by a Fiber-Mutant Adenoviral Vector. Mol. Ther. 7 (3), 354–365. 10.1016/S1525-0016(02)00062-X 12668131

[B101] Tyler M. A. Ulasov I. V. Sonabend A. M. Nandi S. Han Y. Marler S. (2009). Neural Stem Cells Target Intracranial Glioma to Deliver an Oncolytic Adenovirus *In Vivo* . Gene Ther. 16 (2), 262–278. 10.1038/gt.2008.165 19078993 PMC2642530

[B102] Uchida K. Nakajima H. Hirai T. Yayama T. Chen K. Guerrero A. R. (2012). The Retrograde Delivery of Adenovirus Vector Carrying the Gene for Brain-Derived Neurotrophic Factor Protects Neurons and Oligodendrocytes from Apoptosis in the Chronically Compressed Spinal Cord of Twy/twy Mice. Spine 37 (26), 2125–2135. 10.1097/BRS.0b013e3182600ef7 22648027

[B103] Uchida K. Nakajima H. Inukai T. Takamura T. Kobayashi S. Furukawa S. (2008). Adenovirus‐mediated Retrograde Transfer of Neurotrophin‐3 Gene Enhances Survival of Anterior Horn Neurons of Twy/twy Mice with Chronic Mechanical Compression of the Spinal Cord. J. Neurosci. Res. 86 (8), 1789–1800. 10.1002/jnr.21627 18253945

[B104] Wang J. M. Zeng Y. S. Wu J. L. Li Y. Teng Y. D. (2011a). Cograft of Neural Stem Cells and Schwann Cells Overexpressing TrkC and Neurotrophin-3 Respectively after Rat Spinal Cord Transection. Biomaterials 32 (30), 7454–7468. 10.1016/j.biomaterials.2011.06.036 21783247

[B105] Wang X. Smith G. M. Xu X. M. (2011b). Preferential and Bidirectional Labeling of the Rubrospinal Tract with Adenovirus-GFP for Monitoring normal and Injured Axons. J. neurotrauma 28 (4), 635–647. 10.1089/neu.2010.1566 21299337 PMC3070148

[B106] Wang X. Xu X. M. (2014). Long-term Survival, Axonal Growth-Promotion, and Myelination of Schwann Cells Grafted into Contused Spinal Cord in Adult Rats. Exp. Neurol. 261, 308–319. 10.1016/j.expneurol.2014.05.022 24873728 PMC4205953

[B107] Watanabe T. S. Ohtori S. Koda M. Aoki Y. Doya H. Shirasawa H. (2006). Adenoviral Gene Transfer in the Peripheral Nervous System. J. Orthopaedic Sci. 11 (1), 64. 10.1007/s00776-005-0971-z 16437351

[B108] Xu G. Jiang X. D. Xu Y. Zhang J. Huang F. H. Chen Z. Z. (2009). Adenoviral-mediated Interleukin-18 Expression in Mesenchymal Stem Cells Effectively Suppresses the Growth of Glioma in Rats. Cel Biol. Int. 33 (4), 466–474. 10.1016/j.cellbi.2008.07.023 18725309

[B109] Yu B. Zhou Y. Wu H. Wang Z. Zhan Y. Feng X. (2012). Seroprevalence of Neutralizing Antibodies to Human Adenovirus Type 5 in Healthy Adults in China. J. Med. Virol. 84 (9), 1408–1414. 10.1002/jmv.23325 22825819

[B110] Zaiss A. K. Liu Q. Bowen G. P. Wong N. C. Bartlett J. S. Muruve D. A. (2002). Differential Activation of Innate Immune Responses by Adenovirus and Adeno-Associated Virus Vectors. J. Virol. 76 (9), 4580–4590. 10.1128/JVI.76.9.4580-4590.2002 11932423 PMC155101

[B111] Zhang W. Yan Q. Zeng Y. S. Zhang X. B. Xiong Y. Wang J. M. (2010). Implantation of Adult Bone Marrow-Derived Mesenchymal Stem Cells Transfected with the Neurotrophin-3 Gene and Pretreated with Retinoic Acid in Completely Transected Spinal Cord. Brain Res. 1359, 256–271. 10.1016/j.brainres.2010.08.072 20816761

[B112] Zhang W. Zeng Y. S. Zhang X. B. Wang J. M. Chen S. J. (2006). Combination of Adenoviral Vector-Mediated Neurotrophin-3 Gene Transfer and Retinoic Acid Promotes Adult Bone Marrow Cells to Differentiate into Neuronal Phenotypes. Neurosci. Lett. 408 (2), 98–103. 10.1016/j.neulet.2006.08.079 16996685

[B113] Zhang X. Zeng Y. Zhang W. Wang J. Wu J. Li J. (2007). Co-transplantation of Neural Stem Cells and NT-3-Overexpressing Schwann Cells in Transected Spinal Cord. J. neurotrauma 24 (12), 1863–1877. 10.1089/neu.2007.0334 18159998

[B114] Zhao T. Li Y. Dai X. Wang J. Qi Y. Wang J. (2012). Effects of Retrograde Gene Transfer of Brain-Derived Neurotrophic Factor in the Rostral Spinal Cord of a Compression Model in Rat. Mol. Biol. Rep. 39 (8), 8045–8051. 10.1007/s11033-012-1651-7 22531936

[B115] Zhou L. Baumgartner B. J. Hill-Felberg S. J. McGowen L. R. Shine H. D. (2003). Neurotrophin-3 Expressed *In Situ* Induces Axonal Plasticity in the Adult Injured Spinal Cord. J. Neurosci. 23 (4), 1424–1431. 10.1523/JNEUROSCI.23-04-01424.2003 12598631 PMC6742279

[B116] Zhou L. Shine H. D. (2003). Neurotrophic Factors Expressed in Both Cortex and Spinal Cord Induce Axonal Plasticity after Spinal Cord Injury. J. Neurosci. Res. 74 (2), 221–226. 10.1002/jnr.10718 14515351

[B117] Ziv Y. Avidan H. Pluchino S. Martino G. Schwartz M. (2006). Synergy between Immune Cells and Adult Neural Stem/progenitor Cells Promotes Functional Recovery from Spinal Cord Injury. Proc. Natl. Acad. Sci. 103 (35), 13174–13179. 10.1073/pnas.0603747103 16938843 PMC1559772

